# Impact of Prolonged COVID-19 Lockdown on Body Mass Index, Eating Habits, and Physical Activity of University Students in Bangladesh: A Web-Based Cross-Sectional Study

**DOI:** 10.3389/fnut.2022.873105

**Published:** 2022-05-20

**Authors:** Md. Jamal Hossain, Foyez Ahmmed, Md. Robin Khan, Parisa Tamannur Rashid, Sorif Hossain, Md. Oliullah Rafi, Md. Rabiul Islam, Saikat Mitra, Talha Bin Emran, Fahadul Islam, Morshed Alam, Md. Moklesur Rahman Sarker, Isa Naina Mohamed

**Affiliations:** ^1^Department of Pharmacy, State University of Bangladesh, Dhaka, Bangladesh; ^2^Department of Statistics, Comilla University, Cumilla, Bangladesh; ^3^Bangladesh Reference Institute for Chemical Measurements, Dhaka, Bangladesh; ^4^Department of Pharmacy, East West University, Dhaka, Bangladesh; ^5^Institute of Statistical Research and Training, University of Dhaka, Dhaka, Bangladesh; ^6^Department of Statistics, Noakhali Science and Technology University, Noakhali, Bangladesh; ^7^Department of Genetic Engineering and Biotechnology, Jashore University of Science and Technology, Jashore, Bangladesh; ^8^Department of Pharmacy, University of Asia Pacific, Dhaka, Bangladesh; ^9^Department of Pharmacy, Faculty of Pharmacy, University of Dhaka, Dhaka, Bangladesh; ^10^Department of Pharmacy, BGC Trust University Bangladesh, Chittagong, Bangladesh; ^11^Department of Pharmacy, Faculty of Allied Health Sciences, Daffodil International University, Dhaka, Bangladesh; ^12^Institute of Education and Research, Jagannath University, Dhaka, Bangladesh; ^13^Pharmacology Department, Medical Faculty, Universiti Kebangsaan Malaysia (The National University of Malaysia), Kuala Lumpur, Malaysia

**Keywords:** obesity, overweight, eating behaviors, physical inactivity, cross-sectional web-based study, Bangladeshi university students

## Abstract

**Objectives:**

This current study aims to assess the prevalence and factors associated with body mass index (BMI), dietary patterns, and the extent of physical activities among university students following the prolonged coronavirus disease 2019 (COVID-19) lockdown in Bangladesh.

**Methods:**

A cross-sectional web-based survey was conducted between July 10 to August 10, 2021, through a pre-designed Google Form to collect the data from Bangladeshi university students (age: ≥18 years). Informed consent was electronically obtained from each participant, and a simple snowball technique was employed during the sampling. Frequency and percentage distribution, paired *t*-test, chi-square [χ^2^] test, and multinomial and binary logistic regression analyses were consecutively applied to analyze the collected data.

**Results:**

Among the total participants (*n* = 1,602), 45.1% were female and 55.6% were 22–25 years' age group students. The BMI (mean ± standard deviation, SD) during the COVID-19 lockdown was 23.52 ± 7.68 kg/m^2^, which was 22.77 ± 4.11 kg/m^2^ during the pre-lockdown period (mean difference = 0.753; *p* < 0.001). The multinomial logistic regression analysis found a significant impact of gender [male vs. female: adjusted relative risk ratio (RRR) = 1.448; 95% confidence interval (CI) = 1.022, 2.053; *p* = 0.037], age (years) (<22 vs. >25: RRR =0.389, 95% CI = 0.213,0.710; *p* = 0.002, and 22–25 vs. >25: RRR = 0.473, 95% CI = 0.290, 0.772; *p* = 0.003), monthly family income (BDT) (<25,000 vs. >50,000: RRR = 0.525, 95% CI = 0.334,0.826; *p* = 0.005), university type (public vs. private: RRR = 0.540, 95% CI = 0.369, 0.791; *p* = 0.002), eating larger meals/snacks (increased vs. unchanged: RRR = 2.401, 95% CI = 1.597, 3.610; *p* < 0.001 and decreased vs. unchanged: RRR = 1.893, 95% CI = 1.218, 2.942; *p* = 0.005), and verbally or physically abuse (yes vs. no: RRR = 1.438, 95% CI = 0.977, 2.116; *p* = 0.066) on obesity during COVID-19 pandemic. Besides, the female students and those who have constant eating habits, were more likely to be underweight. Additionally, the binary logistic regression analysis found that the students from private universities [others vs. private: adjusted odds ratio (AOR) = 0.461, 95% CI = 0.313, 0.680; *p* < 0.001], urban areas (urban vs. rural: AOR = 1.451, 95% CI = 1.165, 1.806; *p* = 0.001), wealthier families (<25,000 BDT vs. >50,000 BDT: AOR = 0.727, 95% CI = 0.540, 0.979; *p* = 0.036), and who were taking larger meals/snacks (increased vs. unchanged: AOR = 2.806, 95% CI = 2.190, 3.596; *p* < 0.001) and had conflicts/arguments with others (no vs. yes: AOR = 0.524, 95% CI = 0.418, 0.657; *p* < 0.001), were significantly more physically inactive. Finally, the level of education and smoking habits significantly influenced the eating habits of university students during the extended strict lockdown in Bangladesh.

**Conclusion:**

The current findings would be helpful tools and evidence for local and international public health experts and policymakers to reverse these worsening effects on students mediated by the prolonged lockdown. Several effective plans, programs, and combined attempts must be earnestly implemented to promote a smooth academic and daily life.

## Introduction

Since its inception back in December 2019, the novel coronavirus disease (COVID-19), caused by a deadly and pathogenic severe acute respiratory syndrome coronavirus 2 (SARS-CoV-2), has been continuing to bring havoc to our civilization, impacting not only the individual lives but also the global systems ranging from trades to national policies (1). Considering the calamitous worldwide situation with a rapid surge of infections, mortality, and morbidity, the World Health Organization (WHO) declared this viral invasion a pandemic on March 11, 2020 (1, 2). Despite the concerted efforts of both the local governments and the international endeavors, the pandemic persists worldwide. On top of that, we are now facing newer challenges due to multiple variants of the virus and a worldwide vaccine shortage, and inequitable distribution. Meanwhile, the notion of “new-normal” trends, including several anti-epidemic strategies, such as social distancing, shutdown or lockdown, isolation, quarantine, and so on, were introduced to counteract the devastating effects and challenges of COVID-19. However, these anti-COVID measures are increasingly impacting all classes of people with sheer frustration, especially university students, with far-reaching effects on their mental, physical, and social lives (2–4).

Being one of the most densely populated countries globally, with around 165 million people, Bangladesh was at a higher risk of inciting the virus among its citizens more swiftly. Besides, the relatively poor health care facilities and institutional capacities have made Bangladesh vulnerable to the COVID-19 outbreak (4). Nevertheless, Bangladesh got the first COVID-19 cases on March 8, 2020, and from March 26, 2020, the Government of Bangladesh declared lockdowns with several restrictive measures and extended these declarations throughout different time slots, so far (5). A nationwide emergency was imposed, including the closure of all categories of educational institutions, in line with the WHO and other local health experts' recommendations, to curb the spread of the virus. However, these prolonged home confinements have negatively influenced socioeconomic status, including physical and mental health behaviors. Remarkably, the students from all grades were affected badly due to the closedown of educational institutions as part of the lockdown measures (6). Around 2.6 million tertiary level (college or university) students in Bangladesh are now suddenly facing an indefinite halt on their academic activities, delaying their graduation and, ultimately, their timely entrance into the job market (6).

The imposed COVID-19 lockdown and its irrefutable regulations significantly influenced human beings' daily routine and activities, including eating habits, dietary choices, and physical and body weight-related behaviors (7). Even though the direct influence of the COVID-19 pandemic on physical activity and body weight is still not clearly defined, a study in Italy found increased consumption of unhealthy food and decreased physical activity among participants during lockdown (8). Furthermore, school closures dramatically reduced exercise or physical activity and prolonged sleep and screen time among children and adolescents (9). Robinson et al. (10) disclosed several alarming results that 56% of UK adults reported more frequent snacks, and 40% of the population exercised less frequently during the COVID-19 shutdown. Besides, the study also revealed that the people fought against numerous barriers to physical activity and healthy eating, where the difficulties in accessing healthy food, lack of motivation, and insufficient mental and social support were associated with higher BMI. According to another study, students from high schools, colleges, and graduate schools showed a significant increase in both BMI (21.8–22.6 kg/m^2^) and obesity (10.5–12.9%; *p* < 0.001) due to the severe COVID-19 lockdown in China (11). Alfawaz et al. (12) reported that the people of Saudi Arabia walking four times/week reduced their walking during COVID-19 home quarantine compared to before COVID-19 (before vs. during = 30.5 vs. 29.1%). In contrast, the prevalence of taking snacks between meals increased significantly during quarantine (27.4 vs. 29.4%, *p* < 0.001). Another study demonstrated that 32% of Saudi Arabian gained higher BMI, whereas 22% lost their body weight during the COVID-19 lockdown. Though the extent of physical activity was reduced, the sleep time and calorie intake increased significantly (*p* = 0.0001) (13). A worldwide cohort study, conducted by Urzeala et al. (14) on participants from 67 countries, concluded that BMI increased significantly during the COVID-19 lockdown.

On the contrary, physical activity significantly decreased by 31.25 and 26.05% for the youth and young (18–35 years) and adults (35–65 years), respectively. In a systematic review, Bakaloudi et al. (15) summarized that 11.1–72.4% of the population over the age of 16 had a significant increase in their body weight, whereas the elderly population over the age of 60 showed a notable loss in body weight (7.2–51.4%) and signs of malnutrition.

Moreover, the COVID-19 outbreak has a diversified impact on smoking. Some people viewed the lockdown as an opportunity to quit smoking, whereas others relied on smoking to cope with stress and emotions (16). A UK study revealed around a 9% increase in smoking or relapsed smoking intensity during the COVID-19 outbreak. This was, however, evidently associated with elevated symptoms of psychological disorders, impaired sleeping, overweight, and reduced quality of life caused by prolonged lockdown (17, 18). The overweight population is more likely to smoke than normal-weight people, and then, again, cessation of smoking may increase body weight (19, 20). Therefore, prolonged lockdown can intensely affect the dynamics of body weight and smoking behavior.

To the best of our knowledge, no study reported the impact of COVID-19 lockdown on BMI, eating habits, and the extent of physical activities among the Bangladeshi population, including university students. However, it is imperative to take prompt measures to mitigate the impact of these lost school hours, learning losses, and, ultimately, the associated mental pressure and physical disturbances they had faced during the lockdowns. So, we examined several sociodemographic factors, including perceived mental health conditions (depression, anxiety, loneliness, and suicidal thoughts), sleep disturbances, physical disturbances, and interpersonal behaviors (conflict with others, physically or verbally abused) potentially associated with BMI, eating habits and physical activity. This, in turn, necessitates a comprehensive assessment of the changes in their lifestyle, if any, to address the issues in the post-lockdown period. Besides, there is strong evidence of changing patterns in their physical and habitual activities, body weight, and other related parameters. Therefore, the current study plans to point out perceived changes between “before” and “during” the COVID-19 lockdown from the viewpoint of BMI, eating habits, and the extent of physical activity among university students in Bangladesh. The study results can further be used by national and international academic professionals, epidemiologists, or policymakers associated with educational institutions to get an in-depth view of the status of their students. Accordingly, effective and targeted actions might be taken for the students to ease the impacts induced by the prolonged social lockdown and, thereby get them back to normal life.

## Methods

### Study Design

A questionnaire-based Google Form was generated and designed for data collection to perform this cross-sectional study. The questionnaire set was drafted under four sections. Section A contained several questions regarding sociodemographic information like gender, age, education level, university type, current living area, monthly family income, and smoking habit. Section B consisted of information regarding BMI (body height and body weight “before COVID-19 lockdown” and “during COVID-19 lockdown” body weight), and section C contained physical activity and eating habit-related questions. Finally, section D had several further mental health-related (for example: sleep disturbance, feeling loneliness, feeling depressed, feeling anxious, and suicidal thoughts), interpersonal (for example conflict/arguments with others, verbally or physically abused), and weight management-related (for example, physical exercise) questions. However, while designing the questionnaire, we considered similar previous studies for adaptation of the questions and all the parameters about our research objectives (10, 21, 22), and several BMI-related parameters were further adjusted in the Bangladesh perspective according to WHO expert consultation (23). Accordingly, 21 questions were finalized and initially drafted in English, which was translated into the Bangla version for the convenience and better understanding of the participants. A professional way for a forward-backward translation process of the questionnaire was adopted with the help of a bilingual expert having good knowledge of medical terminology (24). Moreover, a total of 12 hypotheses have been synthesized to analyze the association between the present study's covariates and outcome variables based on the target group of this study (8–10, 25–29). The study hypothesis development section was described in the Supplementary Material. Besides, to better understand the current study objective and hypothesis, a tentative conceptual framework might be sketched in Supplementary Figure 1.

### Data Collection and Sampling Technique

The students from the leading three categorized universities: public universities governed by the government, private universities conducted by various private organizations, and other universities (various medical colleges or universities equivalent to the government or private colleges run by the government or private organizations) in Bangladesh were targeted for data collection. As we had no contact details of all university students, a simple web-based snowball sampling strategy has been employed to recruit the target samples in this current pandemic situation (2, 24, 30). The designed Google Form link was shared with them through various popular social media platforms (Facebook, Messenger, Instagram, IMO, WhatsApp, and so on) to collect data relevant to this study. The respondents were requested but not mandatorily to further share the link with other students who might meet the eligibility criteria for this survey. A widely used standard equation, *n* = (Z_α/2_)^2^ × [p(1-p)/(d)^2^], was applied for estimating the size of the study sample, where *n* denotes the sample size, and p represents the proportion of the population (here 50% were expected; *p* = 0.5). The Z_α/2_ (1.96) means the normal distribution value at the 5% significance level, and d indicates the standard error at the 5% tolerated level (24). According to the formula, the calculated sample was 384. Hence a total of 1,718 participants participated voluntarily in the survey between July 10 to August 10, 2021, with no financial compensation. However, we had to eliminate 6.7% (*n* = 116) incomplete or partial responses, while cleaning the raw datasheet. Finally, we have analyzed a total of 1,602 participants, which might lead to more comprehensive and reliable study results.

### Eligibility, Ethics, and Approval

University students of Bangladesh (age: 18 years or above), who have internet access who understood the purpose of this study and were willing to take part, were encouraged to respond to the survey. Additionally, the participants, who had clinical symptoms of dementia during participation in the study and had psychological disturbance before COVID-19 lockdown, were immensely requested to avoid responding to the survey. The questionnaire began with a brief introduction regarding the study's objectives, a declaration of respondents' anonymity, instructions on filling out the survey, and sharing the link with other eligible participants. Informed consent from each participant was also collected virtually before the participation. All the collected data were privately and confidentially preserved. Besides, all the guidelines and ethical protocols of the World Medical Declaration of Helsinki were strictly followed in this questionnaire-based survey. Furthermore, the Human Ethics Committee, State University of Bangladesh, has approved all the protocols and procedures of the study and provided an ethical approval number (2021-06-17/SUB/ERC/0004) after a critical revision and evaluation of the research details.

### Independent Variables

In this current analysis, we included several sociodemographic factors, such as gender (male vs. female), age (18 to below 22 years, 22–25, and above 25 years), education level (lower grade-1st/2nd/3rd year vs. higher grade-4th/5th/Master's or above), current living area (urban vs. rural), monthly family income [below 25,000 BDT, 25,000–50,000 BDT, and above 50,000 BDT; 1 USD = 84.48 Bangladeshi Taka (BDT) as of August 22, 2021], university type (private, public, and others), smoking habits (yes vs. no), meal patterns, several physical and psychological parameters, and interpersonal behavioral manifestations. Physical activity, physical exercise, sleep disturbance, and meal patterns were subdivided into three options “increased,” “deceased,” and “unchanged.” At the same time, the psychological parameters (depression, anxiety, loneliness, and suicidal thoughts) and interpersonal behaviors (conflicting with others and physically or verbally abused) were categorized as yes vs. no.

### Assessment of BMI and Other Dependent Variables

In this survey, all the participated participants mandatorily filled their height and two weights of “before COVID-19 lockdown” and “during COVID-19 lockdown.” BMI of each participant was measured from the very well-known ratio of weight (kg)/height (m^2^), and the cut-off scores for Asia and South-Asian countries were adopted to define the underweight (BMI: <18.5 kg/m^2^), normal weight (BMI: ≥18.5–23 kg/m^2^), overweight (BMI: ≥23–27.5 kg/m^2^), and obesity (BMI > 27.5 kg/m^2^) according to the WHO expert consultation (23). Besides, eating a larger amount of meals or snacks (increased, decreased, unchanged) and physical inactivity (increased, decreased, unchanged) were two other dependent variables of this current analysis.

### Statistical Analysis

Descriptive statistics (frequency and percentage distribution) for sociodemographic characteristics of the eligible participants who answered the questionnaire entirely were assessed during univariate analysis. Besides, the mean and standard deviation (SD) were also measured for comparing the BMI of the participants “before” and “during” COVID-19 lockdown. Paired *t*-test was applied to observe whether BMI increased significantly in the “during” social lockdown period compared to the BMI “before” the COVID-19 lockdown. Association of BMI, eating large meals/snacks, and physical activity with different socioeconomic, demographic, psychological, and interpersonal behavior was evaluated using the chi-square (χ^2^) test of association. Multinomial logistic regression was used to find the potential factors associated with various degrees of BMI. In contrast, binary logistic regression was applied to get the factors potentially related to eating larger meals/snacks and physical inactivity among university students in Bangladesh.

### Software

We used software IBM SPSS version 20 to apply the above-mentioned models in our data. We also used software STATA version 15 and ggplot2 package from R version 4.0.5 for drawing graphs.

## Results

### Sociodemographic Characteristics

A total of 1,602 students [average height (mean ± standard deviation, SD) = 164.5 ± 9.5 cm] responded to the questionnaire completely; among them, 51.7% (*n* = 829) and 38.9% (*n* = 623) were from private and public universities, respectively, and 54.95% (*n* = 880) of the total participants were male students (Supplementary Table 1). Most of the students belonged to the age group 22-25 (55.6%; *n* = 890) from the three age categories (18 to below 22 years, 22–25 years, and above 25 years), and around 60.5 % (*n* = 969) of the respondents were living in the urban area. More than half of the participants (55.2%, *n* = 884) were from the lower grade education level (level of schooling: 1st/2nd/3rd year), and most of the students' (40.1%, *n* = 642) monthly family income was between 25,000–50,000 BDT (Bangladeshi Taka; 1 USD = 84.48 BDT as of August 7, 2021). Besides, out of 1,602 Bangladeshi university students, 227 (14.2%) had regularly smoking habits. Likewise, all the demographic variables with their descriptive statistics are summarized in Supplementary Table 1.

About 35.1% of the participants took larger meals/snacks during the COVID-19 lockdown, while 56.4 and 45.1% of the students decreased their physical activity and physical exercise, respectively, compared to their normal days before the lockdown. Besides, 43.8% of the students faced sleep disturbance. Alarmingly, 68.5% of the students felt lonely, and more than 70% of the participants voted that they were suffering from depression and anxiety. More than half of the respondents had suicidal thoughts, while 28.7% (*n* = 459) of the participants were orally or physically abused (Supplementary Table 1).

### Comparison of the Degree of BMI Between “Before” and “During” COVID-19 Lockdown

**Figure 1:**
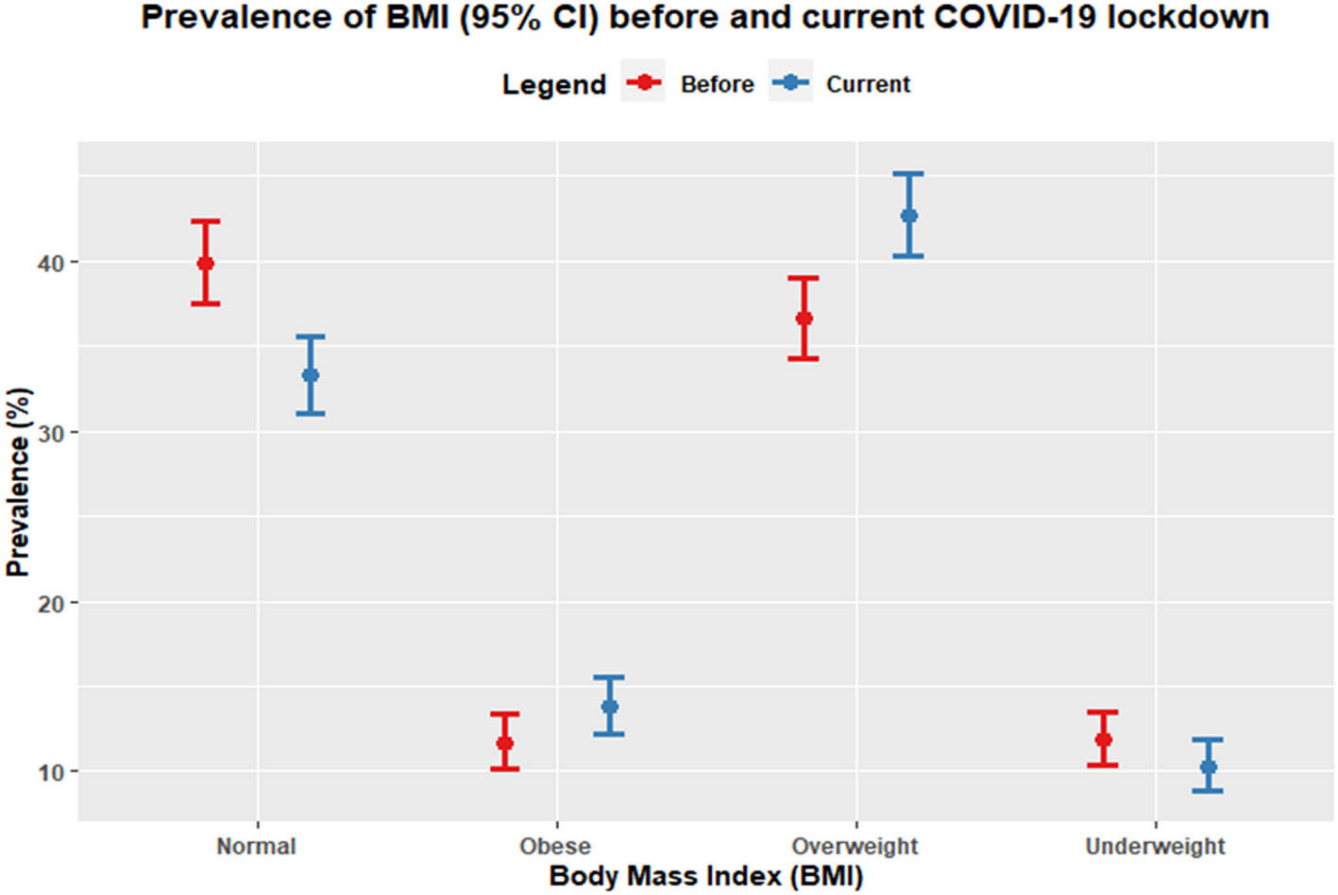
The prevalence of body mass index (BMI) “before” and “current or during” the COVID-19 lockdown among the university students of Bangladesh.

It is noted that 6.4% (during lockdown: 42.7% and before lockdown: 36.3%; Supplementary Table 1) and 2.1% (during lockdown: 13.8% and before lockdown: 11.7%; Supplementary Table 1) of the students gained overweight and obesity, respectively, due to over 1 year of home confinement and closure of educational institution in Bangladesh. On the other hand, due to the prolonged COVID-19 lockdown, significant changes appeared in the degree of BMI among the study participants with an increased mean difference of 0.753 (*p* < 0.001), which is described in Table 1 with *p*-values obtained from paired *t-*test. Before lockdown, there were 39.9% (*n* = 639) of the participants with normal BMI, which has now decreased to 33.3% (*n* = 533). Besides, Figure 1 delineated a significant increasing trend for both overweight and obese during COVID-19 lockdown; in contrast, there was a decreasing trend for both normal BMI and underweight participants.

**Table 1:** Descriptive statistics of body mass index (BMI) before and during COVID-2019 lockdown by different socio-demographic characteristics (p-value obtained from paired t-test).

**Variables**	**Options**	**Body mass index (BMI)**				**Mean difference**	**Paired *t*-test**	***p*-value**
		**Current BMI**		**BMI before COVID-19**				
		**Mean**	**SD**	**Mean**	**SD**			
**Overall**		23.526	7.680	22.772	4.111	0.753	4.42	**<0.001**
Gender	Male	23.993	9.5872	23.0896	3.9517	0.904	3.03	**0.002**
	Female	22.957	4.2842	22.3871	4.2691	0.570	5.33	**<0.001**
Age (years)	<22	23.076	4.4475	22.4844	4.3552	0.592	4.10	**<0.001**
	22–25	23.207	3.9613	22.6815	3.9179	0.525	7.59	**<0.001**
	>25	25.524	16.887	23.6462	4.2197	1.87	1.80	**0.036**
Education level	Lower grade	23.167	4.2921	22.5739	4.3104	0.593	6.07	**<0.001**
	Higher-grade	23.968	10.425	23.0182	3.8410	0.950	2.63	**0.004**
Current living area	Urban	23.435	4.0656	22.8462	4.0763	0.589	7.76	**<0.001**
	Rural	23.666	11.140	22.6609	4.1652	1.005	2.41	**0.007**
Monthly family income (BDT)	<25,000	23.446	11.244	22.2238	3.9703	1.22	2.85	**0.002**
	25,000–50,000	23.378	4.0317	22.8885	4.0317	0.490	6.28	**<0.001**
	>50,000	23.951	4.0939	23.5563	4.3725	0.394	3.37	**<0.001**
University type	Public	23.236	3.9505	22.4564	3.6152	0.780	7.10	**<0.001**
	Private	23.709	9.9152	22.9181	4.4151	0.791	2.49	**<0.001**
	Others	23.719	4.6756	23.2861	4.2356	0.433	2.59	**<0.001**
Smoking habit	Yes	23.823	3.7346	23.0941	3.8338	0.729	3.78	**<0.001**
	No	23.477	8.1505	22.7200	4.1543	0.757	3.86	**<0.001**

**Figure 2:**
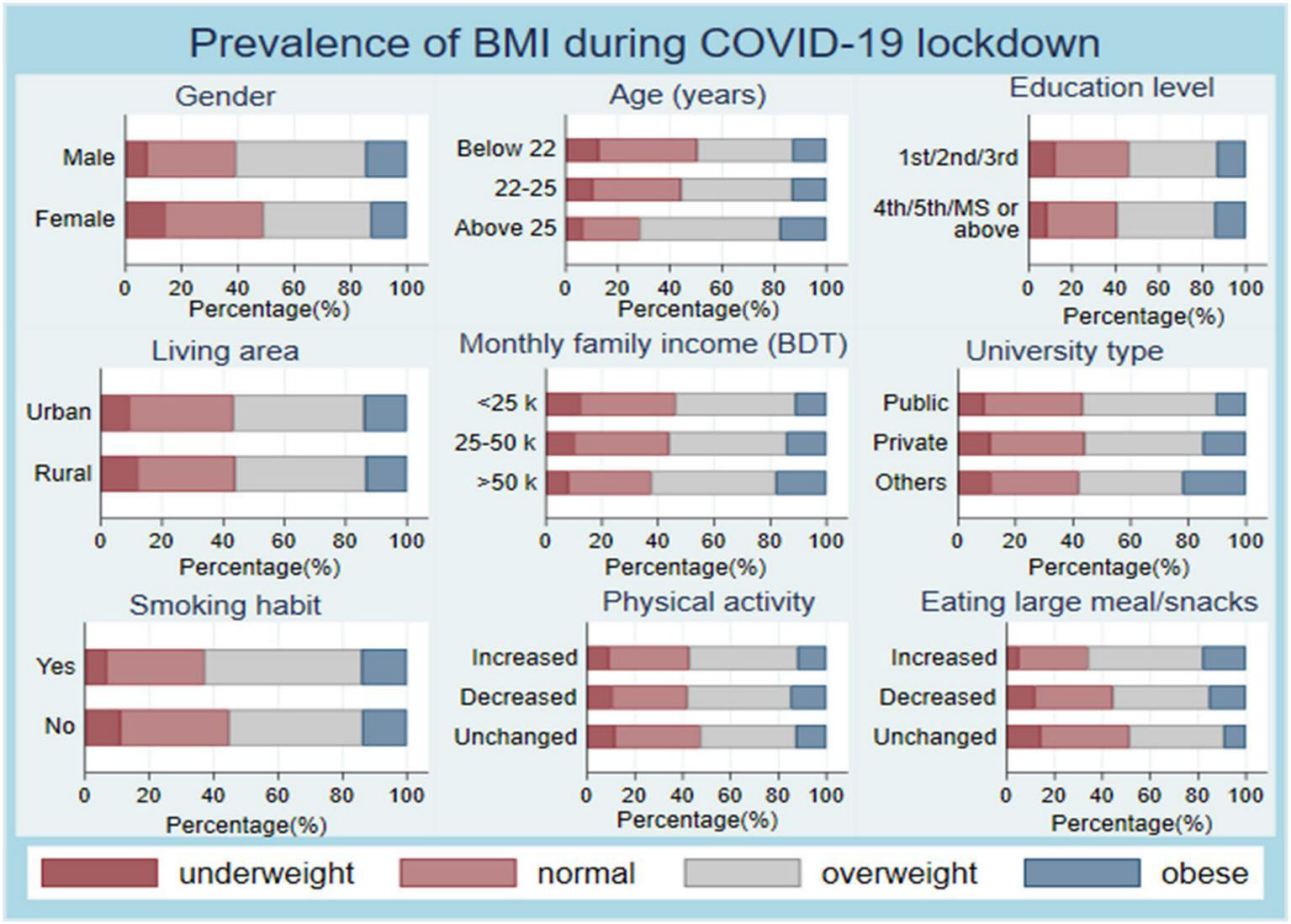
The prevalence of BMI among several sociodemographic groups of university students in Bangladesh during the prolonged COVID-19 lockdown.

The BMI increased significantly within the lockdown, with a mean difference of 0.904 (*p* = 0.002) in male participants. In contrast, the increase of BMI was soaring at the highest rate for the > 25 years age group among the three age groups with a mean difference of 1.87 (*p* = 0.036). Consequently, the upsurge of BMI during the lockdown was the highest rate among higher-grade (4th/5th/Master's or above) university students with a mean difference of 0.950 (*p* = 0.004), while the private university students showed the most elevation of BMI following the prolonged lockdown (mean difference = 0.791, *p* < 0.001). Moreover, the rural and urban students exhibited higher BMI during lockdown than the before lockdown, with a mean difference of 1.005 (mean ± SD: 23.666 ± 11.14 vs. 22.6609 ± 4.1652; *p* = 0.007) and 0.589 (23.435 ± 4.0656 vs. 22.8462 ± 4.0763; *p* < 0.001), respectively. Furthermore, participants from three categories according to monthly family income (<25,000 BDT, 25,000–50,000 BDT, and >50,000 BDT) displayed increased BMI with mean difference 1.22 (*p* = 0.002), 0.490 (*p* < 0.001), and 0.394 (*p* < 0.001), respectively. Public and private university students showed elevated BMI with a unique mean difference of almost 0.8 (*p* < 0.001), whereas the students from other institutions (government colleges or various medical colleges) showed a small increased mean difference of 0.433 (*p* < 0.001). Finally, both smokers and non-smokers showed 0.729 (*p* < 0.001) and 0.757 (*p* < 0.001) mean differences in BMI, respectively, between before lockdown and during lockdown (Table 1). Besides, the prevalence of BMI among the students from various sociodemographic categories during the COVID-19 lockdown was illustrated in Figure 2.

### Chi-Square (χ^2^) Analysis

#### The Degree of BMI and Associated Potential Factors

The χ^2^ analysis was performed to investigate the association of different sociodemographic, physical, psychological, and interpersonal behavioral factors with the degree of BMI among Bangladeshi university students, and the findings were tabulated in Table 2. The χ^2^ test identified a significant association with all the listed variables with the degree of BMI, except the current living area of the participants, physical activity, and mental disturbance parameters (*p* > 0.05).

**Table 2:** Chi-square (χ^2^) test for finding potential association of sociodemographic and several physical, psychological, and interpersonal behavioral factors with the degree of body mass index (BMI) among university students following prolonged COVID-19 lockdown in Bangladesh (*N* = 1,602).

**Variables**	**Categories**	**Current BMI after prolonged COVID 19 lockdown**								***p*-value**
		**Underweight**		**Normal**		**Overweight**		**Obese**		
		***N***	**%**	***N***	**%**	***N***	**%**	***N***	**%**	
Gender	Male	66	7.5	279	31.7	406	46.1	129	14.7	**<0.001**
	Female	98	13.6	254	35.2	278	38.5	92	12.7	
Age (years)	<22	58	12.5	176	37.8	171	36.8	60	12.9	**<0.001**
	22–25	91	10.2	302	33.9	380	42.7	117	13.1	
	>25	15	6.1	55	22.3	133	53.8	44	17.8	
Education level (level of schooling)	Lower grade	106	12.0	300	33.9	360	40.7	118	13.3	**0.041**
	Higher-grade	58	8.1	233	32.5	324	45.1	103	14.3	
Current living area	Urban	89	9.2	331	34.2	413	42.6	136	14.0	0.342
	Rural	75	11.8	202	31.9	271	42.8	85	13.4	
Monthly family income (BDT)	<25,000	74	11.9	213	34.4	264	42.6	69	11.1	**0.042**
	25,000–50,000	63	9.8	219	34.1	269	41.9	91	14.2	
	>50,000	27	7.9	101	29.7	151	44.4	61	17.9	
University type	Public	56	9.0	214	34.3	289	46.4	64	10.3	**0.003**
	Private	91	11.0	273	32.9	341	41.1	124	15.0	
	Others	17	11.3	46	30.7	54	36.0	33	22.0	
Smoking habit	Yes	15	6.6	69	30.4	111	48.9	32	14.1	**0.091**
	No	149	10.8	464	33.7	573	41.7	189	13.7	
Eating large meals or snacks	Increased	28	5.0	163	29.0	270	48.0	102	18.1	**<0.001**
	Decreased	45	11.7	125	32.6	155	40.4	59	15.4	
	Unchanged	91	13.9	245	37.4	259	39.5	60	9.2	
Physical exercise	Increased	20	6.5	102	33.1	154	50.0	32	10.4	**<0.001**
	Decreased	66	9.1	218	30.2	323	44.7	116	16.0	
	Unchanged	78	13.7	213	37.3	207	36.3	73	12.8	
Physical activity	Increased	24	8.8	93	33.9	124	45.3	33	12.0	0.479
	Decreased	92	10.2	287	31.8	390	43.2	134	14.8	
	Unchanged	48	11.3	153	36.0	170	40.0	54	12.7	
Sleep disturbance	Increased	73	10.4	232	33.1	296	42.2	100	14.3	0.927
	Decreased	40	10.4	126	32.6	162	42.0	58	15.0	
	Unchanged	51	9.9	175	34.0	226	43.9	63	12.2	
Feeling lonely	Yes	116	10.6	354	32.2	470	42.8	158	14.4	0.497
	No	48	9.5	179	35.5	214	42.5	63	12.5	
Feeling depressed	Yes	124	10.8	371	32.3	493	42.9	161	14.0	0.461
	No	40	8.8	162	35.8	191	42.2	60	13.2	
Feeling anxious	Yes	124	10.7	374	32.2	499	43.0	164	14.1	0.451
	No	40	9.1	159	36.1	185	42.0	57	12.9	
Suicidal thoughts	Yes	35	10.3	113	33.2	125	36.8	67	19.7	**0.002**
	No	129	10.2	420	33.3	559	44.3	154	12.2	
Conflict/argument with others	Yes	79	9.2	281	32.9	354	41.4	141	16.5	**0.007**
	No	85	11.4	252	33.7	330	44.2	80	10.7	
Verbally or physically abused	Yes	41	8.9	146	31.8	186	40.5	86	18.7	**0.004**
	No	123	10.8	387	33.9	498	43.6	135	11.8	

Numerically, the male students showed 7.6% (46.1 vs. 38.5%; *p* < 0.001) and 2% (14.7 vs. 12.7%; *p* < 0.001) more likely to be overweight and obese, respectively, compared to the female students. The students with > 25 years exhibited significantly more overweight (53.8%) and obesity (17.8%) compared to the other two age groups of <22 years (overweight = 36.8% and obese = 12.9%) and 22–25 years (overweight = 42.7% and obesity = 13.1%). Besides, higher grade students were 4.4% (45.1 vs. 40.7%; *p* = 0.041), and 1% (14.3 vs. 13.3%; *p* = 0.041) more overweight and obese, respectively, than the lower grade students.

Moreover, the students, who belonged to families with monthly income >50,000 BDT, significantly showed 1.8% (44.4 vs. 42.6%; *p* = 0.042), and 2.5% (44.4 vs. 41.9%; *p* = 0.042) more overweight and 6.8% (17.9 vs. 11.1%; *p* = 0.042), and 3.7% (17.9 vs. 14.2%; *p* = 0.042) more obese compared to the rest two counter groups (<25,000 and 25,000–50,000 BDT), respectively. Similarly public and private university students significantly showed 10.4% (36 vs. 46.4%; *p* = 0.003) and 5.1% (36 vs. 41.1%; *p* = 0.003) less overweight and 11.7 (10.3% vs. 22%; *p* = 0.003), and 7% (15 vs. 22%; *p* = 0.003) less obesity, respectively, compared to the other university students after the prolonged lockdown. Besides, participants taking larger meals or snacks were 7.6% (48 vs. 40.4%; *p* < 0.001) and 8.5% (48 vs. 39.5%; *p* < 0.001) more overweight and 2.7% (18.1 vs. 15.4%; *p* < 0.001) and 8.9% (18.1 vs. 9.2%; *p* < 0.001) more obese compared to the participants with decreased and unchanged food habit, respectively. The effect of smoking is also evident from the data with 7.2% (48.9 vs. 41.7%; *p* = 0.091) and 0.4% (14.1 vs. 13.7%; *p* = 0.091) more overweight and obese participants in the smokers than the non-smoker participants. Besides, participants with suicidal thoughts showed significantly 7.5% (19.7 vs. 12.2%; *p* = 0.002) more obese. Finally, verbally or physically abused students were 6.9% (18.7 vs. 11.8%; *p* = 0.004) more obese than the other participants, and students who involved conflicting or arguments with others had 5.8% (16.5 vs. 10.7%; *p* = 0.007) more obesity than others.

#### Eating Habits and Associated Potential Factors

The results from the χ^2^ test (Table 3) demonstrated that age, education level, and smoking habit significantly influenced the outcome variable, which is “eating large meals/snacks,” among the university students following the prolonged COVID-19 lockdown in Bangladesh. Students from the age group above 25 years exerted 11.1% (40.1 vs. 29%, *p* = 0.014) and 3.1% (40.1 vs. 37%, *p* = 0.014) more increased eating of larger meals/snacks than the other two age groups (>22 and 22–25 years, respectively), whereas 1% (25.2 vs. 24.2%, *p* = 0.014) and 4.1% (25.2 vs. 21.1%, *p* = 0.014) of students from below 22 years age group showed more decreased eating of larger meals/snacks than the other two age groups (22–25 and >25 years, respectively). Besides, 2.1% (24.9 vs. 22.8%, *p* < 0.001) of students in lower grade levels and 13.5% (46.7 vs. 33.2%, *p* < 0.001) of smokers decrease their consumption of larger meals/snacks compared to their counter groups, respectively, during the COVID-19 lockdown in Bangladesh.

**Table 3:** Chi-square (χ^2^) test for finding the potential association of sociodemographic factors impacting eating larger meals/snacks and physical activity among university students following prolonged lockdown in Bangladesh (*N* = 1,602).

**Variables**	**Categories**	**Eating larger meals or snacks**							**Physical activity**						
		**Increased**		**Decreased**		**Unchanged**		***p*-value**	**Increased**		**Decreased**		**Unchanged**		***p*-value**
		***N***	**%**	***N***	**%**	***N***	**%**		***N***	**%**	***N***	**%**	***N***	**%**	
Gender	Male	304	34.5	209	23.8	367	41.7	0.757	170	19.3	473	53.8	237	26.9	**0.018**
	Female	259	35.9	175	24.2	288	39.9		104	14.4	430	59.6	188	26.0	
Age (years)	<22	135	29.0	117	25.2	213	45.8	**0.014**	79	17.0	257	55.3	129	27.7	0.598
	22–25	329	37.0	215	24.2	346	38.9		149	16.7	516	58.0	225	25.3	
	>25	99	40.1	52	21.1	96	38.9		46	18.6	130	52.6	71	28.7	
Education level	Lower grade	268	30.3	220	24.9	396	44.8	**<0.001**	152	17.2	499	56.4	233	26.4	0.983
	Higher-grade	295	41.1	164	22.8	259	36.1		122	17.0	404	56.3	192	26.7	
Current living area	Urban	331	34.2	237	24.5	401	41.4	0.586	152	15.7	588	60.7	229	23.6	**<0.001**
	Rural	232	36.7	147	23.2	254	40.1		122	19.3	315	49.8	196	31.0	
Monthly family income (BDT)	<25,000	209	33.7	145	23.4	266	42.9	0.107	109	17.6	301	48.5	210	33.9	**<0.001**
	25,000–50,000	248	38.6	154	24.0	240	37.4		107	16.7	394	61.4	141	22.0	
	>50,000	106	31.2	85	25.0	149	43.8		58	17.1	208	61.2	74	21.8	
University type	Public	241	38.7	142	22.8	240	38.5	0.127	109	17.5	363	58.3	151	24.2	**<0.001**
	Private	266	32.1	208	25.1	355	42.8		135	16.3	482	58.1	212	25.6	
	Others	56	37.3	34	22.7	60	40.0		30	20.0	58	38.7	62	41.3	
Smoking habits	Yes	106	46.7	50	22.0	71	31.3	**<0.001**	35	15.4	127	55.9	65	28.6	0.641
	No	457	33.2	334	24.3	584	42.5		239	17.4	776	56.4	360	26.2	
Conflict/arguments	Yes								140	16.4	551	64.4	164	19.2	**<0.001**
	No								134	17.9	352	47.1	261	34.9	
Verbally/physically abused	Yes								90	19.6	264	57.5	105	22.9	**0.057**
	No								184	16.1	639	55.9	320	28.0	
Large meals/snacks	Increased								89	15.8	393	69.8	81	14.4	**<0.001**
	Decreased								89	23.2	221	57.6	74	19.3	
	Unchanged								96	14.7	289	44.1	270	41.2	

#### Physical Activity and Associated Potential Factors

It is clear from Table 3 that all the variables, except age, education level, and smoking habit, were significantly associated with the university students' physical activity during the COVID-19 lockdown in Bangladesh. Male students showed 4.9% (19.3 vs. 14.4%, *p* = 0.018) more increased physical activity than the female students. Contrary, female students exhibited 5.8% more decreased physical activity than male students. Similarly, rural participants showed 3.6% (19.3 vs. 15.7%, *p* < 0.001) more increased physical activity compared to the urban participants, whereas urban participants exerted 10.9% (60.7 vs. 49.8%, *p* < 0.001) more decreased physical activity compared to the rural participants. Besides, monthly family income and university type have significantly influenced the status of physical activity of the current university students. Notably, the participants involved in conflict/argument with others and abused verbally or physically were more likely to have decreased physical activity by 17.3% (64.4 vs. 47.1%, *p* < 0.001) and 1.6% (57.5 vs. 55.9%, *p* = 0.057), respectively, than their counter groups. Besides, the larger meals/snacks takers manifested 25.7% (69.8 vs. 44.1%, *p* < 0.001) more decreased physical activity than the group of unchanged amount meals/snacks takers.

### Regression Analysis

#### Multinomial Regression for the Degree of BMI

Multinomial logistic regression analysis was carried out to assess the significant association of factors with underweight, overweight, and obese respondents compared to with normal weight after adjusting for other factors, and the outcomes were abridged in Table 4.

**Table 4:** Adjusted relative risk ratio (RRR) from multinomial logistic regression analysis for underweight, overweight, and obese respondents in comparison with normal weight respondents of university students following prolonged COVID-19 lockdown in Bangladesh.

**Covariates**	**Categories**	**Underweight**				**Overweight**				**Obesity**			
		**RRR**	***p*-value**	**95% CI**		**RRR**	***p*-value**	**95% CI**		**RRR**	***p*-value**	**95% CI**	
				**Lower**	**Upper**			**Lower**	**Upper**			**Lower**	**upper**
Gender	Male	0.590	**0.008**	0.400	0.869	1.225	0.110	0.955	1.571	1.448	**0.037**	1.022	2.053
	Female^R^												
Age (years)	<22	0.732	0.413	0.348	1.543	0.352	**<0.001**	0.224	0.552	0.389	**0.002**	0.213	0.710
	22–25	0.900	0.752	0.470	1.724	0.470	**<0.001**	0.324	0.681	0.473	**0.003**	0.290	0.772
	>25^R^												
Education level	Lower grade	1.430	0.103	0.931	2.197	1.240	0.123	0.943	1.631	1.228	0.294	0.837	1.803
	Higher-grade^R^												
Monthly family income (BDT)	<25,000	1.486	0.142	0.876	2.520	0.889	0.488	0.638	1.239	0.525	**0.005**	0.334	0.826
	25,000–50,000	1.138	0.623	0.678	1.911	0.810	0.193	0.589	1.112	0.688	**0.079**	0.454	1.044
	>50,000^R^												
University type	Public	0.821	0.341	0.548	1.231	0.993	0.959	0.769	1.283	0.540	**0.002**	0.369	0.791
	Others	1.106	0.758	0.583	2.100	0.882	0.585	0.563	1.383	1.470	0.160	0.859	2.517
	Private^R^												
Smoking status	Yes	0.924	0.804	0.494	1.726	1.104	0.577	0.779	1.564	0.836	0.475	0.513	1.365
	No^R^												
Eating larger meals or snacks	Increased	0.495	**0.005**	0.302	0.813	1.476	**0.007**	1.112	1.959	2.401	**<0.001**	1.597	3.610
	Decreased	1.034	0.881	0.668	1.601	1.111	0.502	0.817	1.511	1.893	**0.005**	1.218	2.942
	Unchanged^R^												
Physical exercise	Increased	0.637	0.117	0.362	1.120	1.474	**0.021**	1.059	2.050	0.702	0.166	0.425	1.159
	Decreased	0.998	0.992	0.666	1.495	1.451	**0.008**	1.102	1.911	1.174	0.406	0.804	1.716
	Unchanged^R^												
Suicidal thoughts	Yes	1.066	0.787	0.669	1.700	0.767	0.094	0.563	1.047	1.296	0.197	0.874	1.920
	No^R^												
Conflict/arguments with others	Yes	0.910	0.635	0.615	1.345	0.922	0.531	0.716	1.188	1.226	0.271	0.853	1.763
	No^R^												
Verbally or physically abused	Yes	0.953	0.840	0.601	1.512	0.968	0.828	0.722	1.297	1.438	**0.066**	0.977	2.116
	No^R^												

R *Reference category*.

The female students were significantly more likely to be risky of being underweight than male students [male vs. female: RRR = 0.590, 95% confidence interval (CI): 0.400, 0.869; *p* = 0.008]. In contrast, the male students were around 1.5 times more likely to be risky for being obese compared to the female students (male vs. female: RRR = 1.448, 95% CI: 1.022, 2.053; *p* = 0.037), respectively. The higher age category (>25 years) was significantly more likely to be risky for being both overweight and obese than the lower age categories (<22 years and 22–25 years). Notably, the students came from the families with monthly income < 25,000 BDT and 25,000–50,000 BDT were 48% (RRR = 0.525, 95% CI: 0.334, 0.826; *p* = 0.005) and 32% (RRR = 0.688, 95% CI: 0.454, 1.044; *p* = 0.079) less risky for being obese than the students from the families with monthly income more than 50,000 BDT. Besides, the public university students reported that they were 46% (95% CI: 0.369, 0.791; *p* = 0.002) less likely to have a chance obese than the private university students. Finally, verbally or physically abused students showed 1.4 (95% CI: 0.977, 2.116; *p* = 0.066) times higher risk for inclination toward obesity than the students with no abuse. Similarly, physical exercise and suicidal thoughts were significantly associated with overweight risk in multinomial regression analysis among university students in Bangladesh (Table 4).

#### Binomial Regression for Eating Larger Meals/Snacks and Physical Inactivity

A binomial logistic regression analysis was conducted during multivariate analysis to assess the significant association of potential factors with increased eating larger meals or snacks and physical inactivity after adjusting for other factors. These findings are listed in Table 5.

**Table 5:** Logistic regression analysis for finding the potential associated factors with “conflict/arguments with others” and “physically or verbally abused by others” following prolonged COVID-19 lockdown among Bangladeshi university students.

**Covariates**	**Categories**	**Increased eating larger meals/snacks**				**Increased physical inactivity**			
		**AOR**	***p*-value**	**95% CI**		**AOR**	***p*-value**	**95% CI**	
				**Lower**	**Upper**			**Lower**	**Upper**
Gender	Male					0.924	0.467	0.746	1.144
	Female^R^								
Age (years)	<22	0.889	0.542	0.608	1.299				
	22–25	1.054	0.735	0.777	1.430				
	>25^R^								
Education level	Lower grade	0.675	**0.001**	0.530	0.860				
	Higher-grade^R^								
Current living area	Urban					1.451	**0.001**	1.165	1.806
	Rural^R^								
Monthly family income (BDT)	<25,000					0.727	**0.036**	0.540	0.979
	25,000–50,000					1.034	0.816	0.777	1.377
	>50,000^R^								
University type	Public					0.961	0.740	0.762	1.212
	Others					0.461	**<0.001**	0.313	0.680
	Private^R^								
Smoking status	Yes	1.673	**<0.001**	1.255	2.229				
	No^R^								
Eating larger meals or snacks	Increased					2.806	**<0.001**	2.190	3.596
	Decreased					1.638	**<0.001**	1.261	2.129
	Unchanged^R^								
Conflict/arguments with others	No					0.524	**<0.001**	0.418	0.657
	Yes^R^								
Verbally or physically abused	No					1.167	0.236	0.904	1.507
	Yes^R^								
Constant		0.621	**<0.001**			1.009	0.963		

Students in higher grade levels were around 33% (95% CI: 0.530, 0.860; *p* =0.001) less likely to have larger meals/snacks than the lower grade students, and students with smoking habits showed to significantly have larger meals/snacks than the non-smokers (AOR = 1.673, 95% CI: 1.255, 2.229; *p* < 0.001). Urban students were consistently more physically inactive than rural students (AOR = 1.451, 95% CI: 1.165, 1.806; *p* = 0.001), and notably, students from lower monthly income families (<25,000 BDT) were 30% less likely to be inactive than those from higher monthly income families (>50,000 BDT) (95% CI: 0.540, 0.979; *p* = 0.036). Besides, respondents from other universities reported being less engaged in physical in-activities than respondents from private universities (Others university vs. Private: AOR =0.461, 95% CI: 0.313, 0.680; *p* < 0.001). Furthermore, students with increased eating of larger meals/snacks reported being 2.8 (95% CI: 2.190, 3.596; *p* < 0.001) more likely to be engaged in physical inactivity than the students who did not change their eating habits. Finally, students with conflicting or arguments with others showed 48% (95% CI: 0.418, 0.657; *p* < 0.001) less likely to be engaged in inactivity than the other students with no conflicting arguments.

## Discussions

The current study, to the best of our searching experience, aims for the first time to assess the changes in BMI among the university students “before” and “during” the COVID-19 lockdown, as well as to determine the relationship of various sociodemographic, psychological, and interpersonal behaviors with BMI changes. In addition, the research also focused on understanding if there was a link between different sociodemographic characteristics with eating habits and physical activity during the COVID-19 lockdown. The present study revealed that underweight, overweight, and obesity prevalence were 11.9, 36.6, and 11.7%, respectively, before the COVID-19 lockdown, which was enumerated as 10.2, 42.7, and 13.8%, respectively, following prolonged COVID-19 strict lockdown. Besides, the current data exhibited a clear perception of dietary patterns and prevalence of physical inactivity and several significantly influencing factors. However, direct comparisons of these findings may be difficult in the prevailing context due to limited evidence or lack of study on Bangladeshi university students in similar settings.

### Determinants of Overweight, Obesity, and Underweight

The current study findings demonstrated a significant rise (during vs. before = 23.526 ± 7.680 vs. 22.772 ± 4.111; *p* < 0.001) in BMI among the university students following more than a year of educational institution closure and home confinement during COVID-19 lockdown in Bangladesh, which is consistent with earlier reported outcomes in several parts of the world (9, 31, 32). The BMI of all sociodemographic subgroups of the participants increased significantly. However, the BMI of male individuals increased substantially more than that of female participants. A similar study of 368 obese people found that women had lower BMI values than men (28.57 ± 3.89 vs. 30.64 ± 2.87) (33). In terms of risk categorized BMI, the male students were 7.6 and 2% more likely to be overweight and obese than the female students, which is also in line with some previous evidence (33, 34). Besides, the current data showed that students from the older age groups (>25 years) were likely to be more at risk for being overweight and obese than those in the younger age groups (22 years and 22–25 years). This finding was supported by the studies conducted on Bangladeshi (35) and Nepalese women (36), where the lower age group subjects were at a lower risk of being overweight or obese than women in the higher age group. In contrast, according to a study conducted in China, BMI and prevalence of overweight and obesity varied significantly across educational levels, with high-school students (age = 17.5 ± 1.2 years) having the highest BMI (22.7 ± 6.7 kg/m^2^) and the highest prevalence of overweight (26.7%) and obesity (16.1%) than the undergrad (age = 20.6 ± 1.8 years) and graduate (age = 24.6 ± 3.5 years) students (33).

Moreover, the current analyses endorsed that the students from families with higher income (>50,000 BDT) were substantially more at risk of being obese than students from the other two counter groups (<25,000 BDT and 25,000–50,000 BDT). This outcome for the wealth-obesity relationship was consistent with the previous studies (35–41) that the richest were more likely to be obese than the poor. Furthermore, it is evident from the multinomial regression analysis that private university students had more risk of obesity during the prolonged lockdown. It is a widespread belief that most private university students come from wealthier families, and they might have a sedentary lifestyle, more access to energy-dense and processed food, and escape from physical work, which might be primarily responsible for higher BMI (40, 41).

The study also revealed that the students taking larger meals or snacks were likely to be 1.5 and 2.4 times more at risk of being overweight and obese, respectively, compared to those with unchanged food habits. Increased eating can be justified by the feeling of boredom, which may arise from staying home for an extended period (42, 43). Huber et al. reported that the increased amount of food consumption during lockdown was significantly mediated by higher BMI compared to the students with normal BMI (OR = 1.427; 95% CI = 1.032–1.974; *p* = 0.032) (21). Similarly, another study reported an increase in overeating during the lockdown in subjects with a higher BMI (10).

According to χ^2^ analysis, physical activity exerted no significant relationship with overweight or obesity; nevertheless, physical exercise had a significant association with the likelihood of being overweight. Besides, the regression analysis found that the students who reduced their physical activity during the lockdown had a higher risk ratio to be overweight than the students who had unchanged physical activity. In line with several prior studies, higher BMI was associated with lower diet quality and decreased physical labor and exercise during physical mobility restrictions (10, 44, 45). On the other hand, the current data claimed that another group of students also gained a higher risk of being overweight upon increased physical exercise compared to the students with unchanged physical exercise. In those cases, there might occur a sudden weight gain with physical exercise for increased lean muscle mass and muscle fuel, as well as several mental complications that mediate emotional/stress-related overeating, prone to taking snacks after dinner during the pandemic situation (46–48). However, there might be some knowledge gap about the involvement of several drivers of the obesity epidemic during the current lockdown period, such as leisure-time exercise or physical activity.

Additionally, the current data also demonstrated that the students who were physically or verbally abused by others were likely to be 1.4 times more at risk of being obese than their peers. Several studies reported the drastic increase in physical or verbal abuse, sexual harassment, conflict, and overall social stigma, including domestic or social violence, during the COVID-19 period in Bangladesh (49, 50). This interpersonal distress, indirect effects of social networking or social destruction, and stigmatization significantly impact the behaviors and psychosocial stress that might strongly associate overall lifestyle and obesity/weight gain promotors (25, 51, 52).

In terms of being underweight, gender and diet/food consumption patterns are the major influencing factors in the study. The current regression analysis traced that the female students were almost 40% more at risk of being underweight than males. Besides, the students who increased their food consumption were significantly less at risk of being underweight than the students who unchanged their food pattern. The prevalence of underweight among women is higher than among men in South-East Asia and the Pacific regions reported in various previous studies due to biological, environmental, and economic factors that might be triggered in the COVID-19 lockdown period and associated with up to the severe level of malnutrition (36, 53).

Although we found a significant association between smoking habits with alterations in BMI during the χ^2^ analysis of the samples, the regression identified no significant relationship between smoking with overweight/obesity. However, our findings exhibited that both smokers and non-smokers increased BMI due to home confinement. In contrast, smokers had a larger likelihood of being overweight or obese than non-smokers. A previous study conducted on USA college students reported that smoking significantly influenced obesity-enhancing behaviors, such as eating high calories dietary and food consumption while watching television (54).

Moreover, we investigated several psychological parameters like depression, anxiety, loneliness, and sleep disturbance with various degrees of BMI as these mental disorders were drastically increased among university students during the closure of educational institutions (24, 26). Although around 70% of the participants were suffering from these mental complications (sleep disturbance: 43.8%), the current data found no significant association of these parameters with underweight, overweight, or obesity. However, the students who had suicidal thoughts were 7.5% more obese than those who did not have suicidal thoughts during the χ^2^ analysis. However, this factor was not significantly related to overweight/obesity in the multinomial regression analysis. Similarly, a study on UK adults found no significant relationship between mental health decline and higher BMI (10). On the contrary, a study discovered that participants who reported changes in their BMI status were more likely to suffer from depression and anxiety, but not suicidality (32).

### Determinants of Consumption of Larger Meals/Snacks

This lockdown condition makes it difficult to eat a balanced and diversified diet. Limited access to daily grocery shopping, and convenience in online shopping, for example, may lead to a shift away from fresh foods like fruits, vegetables, and seafood to highly processed meals like convenience foods, junk foods, snacks, etc. Furthermore, psychological and emotional reactions to the COVID-19 outbreak enhanced the chance of developing imbalanced eating habits (32). The current study demonstrated that the higher-grade students were more likely to take larger meals/snacks during the lockdown than lower-grade university students. Various studies on Bangladeshi university students reported that higher-grade students suffered more from mental depression than lower-grade students (26). Academic and lockdown-induced psychological stress might affect the eating behaviors among university students (55).

Furthermore, the current evidence exerted that the university students who were regular smokers had a considerably higher tendency (1.7 times) to consume larger meals/snacks than non-smokers. Similarly, Huber et al. (21) reported that the university students who had smoking habits were 11.5% (42 vs. 30.5%; *p* = 0.012) more prone to taking larger meals/snacks than the non-smokers during the COVID-19 lockdown. Though the current study found a significant association between age and eating behaviors, the regression analysis found no significant association between age groups with increased eating larger meals. This prediction was also consistent with some of the previous study's findings (10, 21).

### Determinants of Physical Inactivity

The COVID-19 restrictions have massively impacted physical activities since the early phase of the pandemic and prohibited people from reaching the threshold level of recommended physical movement for being healthy. The current data reported that around half of the students sharply minimized their physical activity and physical exercise. Consistently, Islam et al. (56) reported that 55.3% of the university students in Bangladesh were not engaged in regular physical exercise. In a systematic review of 66 studies, Stockwell et al. (57) disclosed that the participants from 64 studies reduced their physical activity and increased their sedentary manners during the COVID-19 lockdown.

Here, the present regression analysis enumerated almost 1.5 times higher engagement with physical inactivity among the urban students than rural students. Similarly, Rahman et al. (58) previously disseminated that urban people of Bangladesh showed 2.2 times (95% CI = 1.8–2.8; *p* < 0.001) higher physical inactivity and 2.9 times (95% CI = 2.2–3.7; *p* < 0.001) higher sedentary behaviors compared to the village people. Physical and social contextual elements that influence access, availability, and behaviors have a significant role in physical activity participation. The urban regions in Bangladesh are densely inhabited, and the number of COVID-19 cases was relatively higher (59–61). Besides, several factors such as fear of COVID-19 infection, the strict shutdown of almost every place (except emergency needs), including playgrounds and gymnasium, lack of companions to exercise with, loss of willingness to pursue physical activities, and so on, might demotivate the university students to be active in physical labors during the social lockdown (58). Furthermore, the students from higher-income families were significantly more physically inactive compared to the students from lower-income families, which was also consistent with the previous report (participants from upper-class families were three times (95% CI = 2.3–4.0, *p* < 0.001) more likely to be physically inactive than the lower-class participants) (58). The current analysis also found that the students from private universities were substantially more physically inactive during the lockdown than those from “others” category institutions. It is well-known that the students from highly reputed private universities come from wealthier families and their lifestyles are flexible, having more sedentary behaviors than the students from more impoverished families or various colleges under the National University of Bangladesh (24). Besides, Trinh et al. (62) disclosed that the household wealth index significantly influenced physical inactivity (highest vs. lowest household wealth index: OR = 1.86, 95% CI = 1.29–2.66).

Moreover, there was a significant link between eating habits and physical inactivity. The students who increased the larger meals/snacks were 2.8 times more likely to be physically inactive than those who did not change their eating habits. Similarly, several previous studies examined the significant increase in taking larger meals/snacks during COVID-19 home confinement (21, 63), which might be a primary mediating factor for showing more sedentary acts and subsequent physical inactivation (58, 64). The study also observed that the students who decreased their eating amounts were significantly and more physically inactive than those who kept their dietary consumption unchanged. Similarly, Huber et al. (21) stated that the young adults who increased their sports activity decreased their food consumption by 1.9 times than the subjects who kept their food habits constant. A potential explanation might be that many students have passed most of their time on the electronic device screen for gaming, social media chatting, or gossiping with other friends during home confinement; these behaviors might be associated with being more physically inactive (25, 58). Besides, the present analysis found that the students having conflict/arguments with others were more physically inactive than their peers. Social isolation and loneliness increased hostility and extreme anger among young adults, thus, provoking psychoticism (65–67). The abnormal mental status might attribute the current university students embroiled in contradictory debates/conflicts and social violence. Although Robinson et al. (10) reported that many participants increased their social arguments/conflict, the authors did not find any significant association of the factor with physical inactivity.

## Practical Implications

According to the best of our knowledge and searching experience, this unique investigation is the first ever to assess the correlations of several diverse sociodemographic factors with the degree of BMI, eating larger meals/snacks, and physical inactivity of the university students under the context of prolonged lockdown in Bangladesh. The results must be required for government agencies, educational institutions, and epidemiologists to address the BMI-related physiological and behavioral issues among the target students. Besides, the current study pointed out the risk factors of COVID-19 associated with obesity or overweight owing to prolonged confinement in the home followed by physical inactivity. These results obtained from this research can also be employed to understand the BMI status, eating habits, and the extent of physical activities among university students of other countries with identical demographic and socioeconomic conditions. Finally, the observations of this study can have implications in future policymaking involving the university students who would be facing a tough time in the aftermath of a pandemic due to the academic hours that they have lost due to lockdowns.

## Study Limitations

The current analysis is not a flawless investigation with some drawbacks. The most noticeable limitation of this cross-sectional web-based survey is the absence of means to cross-check the quality of the self-reported data provided by the respondents. It was impossible on our part to assess whether the respondents had adequate thoughts before filling the google form unbiasedly. Besides, it is highly likely that students from rural or remote areas with no proper internet connectivity could not participate in the survey, which made the data comparatively less representative of the whole country. The study was conducted inside Bangladesh only; therefore, adequate cautions might be required while interpreting the results for other regions, particularly for the countries with irreconcilable demographic and socioeconomic characteristics. Furthermore, the study did not measure the pre-lockdown weight management behaviors that might be crucial for understanding the weight gain due to movement restrictions. Lastly, the participants had to answer their pre-lockdown weights that might be subject to recall bias. Also, height was collected only during the survey, which may somewhat affect the rigorous inventory of BMI with the time variation.

## Future Research

Some prospective studies might be designed to focus on comparing the significant changes in BMI of students amid the COVID-19 lockdown with other age groups of people. Besides, there are several scopes for the current research to advance and explore novel dynamics between all the recommended weight management behaviors and health concerns, including obesity among university students. The ongoing pandemic may further impose lockdown measures from time to time, and, thus, the findings of the current research can be explored in the long term to assess the impact on university students. The analysis can also be expanded by incorporating more participants of diversified demographic features to establish more reproducible research findings. Notably, the current research for policy reinforcement must be emphasized and intensified to vigorously assess the obesity influencing factors that might have a significant association with physical activities and regular dietary patterns among all the vulnerable groups, especially women and students.

## Conclusion

The current analysis identified that the extended COVID-19 lockdown has profoundly impacted the eating behaviors and the extent of physical activities and BMI of university students in Bangladesh. The study inferred that the prevalence of overweight/obesity and underweight has disproportionately risen, and the degree of BMI has been substantially influenced by several demographic and socioeconomic factors, including eating habits and physical activity. Besides, the closure of educational institutions and stringent movement restrictions significantly increased food consumption and physical inactivity. In the light of the current findings and evidence, several concerted attempts, plans, and programs must be warranted by epidemiologists, institutional administration, and government policymakers to employ weight management behaviors, balanced diets, and appropriate physical activities, including physical exercise in the context of home confinement measures. Apart from professional counseling to promote awareness for avoiding sedentary behaviors, smooth academic activities need to be secured to minimize the study gap due to the extended shutdowns.

## Data Availability Statement

The original contributions presented in the study are included in the article/Supplementary Material, further inquiries can be directed to the corresponding authors.

## Ethics Statement

Informed consent from each participant was also collected virtually before the participation. All the collected data were preserved private and confidential. Besides, all the guidelines and ethical protocols of the World Medical Declaration of Helsinki were strictly followed in this questionnaire-based survey. Furthermore, the Human Ethics Committee, State University of Bangladesh, has approved all the protocols and procedures of the study and provided an ethical approval number (2021-06-17/SUB/ERC/0004) after a critical revision and evaluation of the research details.

## Author Contributions

MH developed the idea of the work. MH and FA designed the study. MH, FA, SH, MR, MI, SM, TE, FI, and MA collected the data. MH and FA cured and analyzed the raw data. MH and FA interpreted the analyzed data. MH, MK, and PR searched the literature and drafted the original manuscript. MH, MS, and IM have made funding acquisitions. MH, MS, TE, and INM critically revised and improved the manuscript. All authors reviewed and approved the final version of the manuscript.

## Conflict of Interest

The authors declare that the research was conducted in the absence of any commercial or financial relationships that could be construed as a potential conflict of interest.

## Publisher's Note

All claims expressed in this article are solely those of the authors and do not necessarily represent those of their affiliated organizations, or those of the publisher, the editors and the reviewers. Any product that may be evaluated in this article, or claim that may be made by its manufacturer, is not guaranteed or endorsed by the publisher.
