# Supplementary material for: Impact of Prolonged COVID-19 Lockdown on Body Mass Index, Eating Habits, and Physical Activity of University Students in Bangladesh: A Web-Based Cross-Sectional Study
Source: Front Nutr. 2022 May 20;9:873105. doi: 10.3389/fnut.2022.873105 (PMC9165530; doi:10.3389/fnut.2022.873105)
Supplement: Supplementary file 1 [file Data_Sheet_1.PDF]

Supplementary file

Title

Impact of prolonged COVID-19 lockdown on body mass index, eating habits, and physical activity of university students in Bangladesh: a web-based cross-sectional study

Study hypotheses (H1 to H12)

COVID-19 steered home confinement has hugely affected the target students, ranging from eating and smoking habits to interpersonal behaviors. A total of 12 hypotheses have been developed in the current study to define the dynamics between these variables with the prolonged lockdowns. Several imposed strict measures during COVID-19 lockdown are closely linked with obesogenic behaviors, particularly physical inactivity and eating larger unhealthy meals that might immensely affect the BMI (1). Besides, the current situation has forced for sitting the same place for a prolonged time with enhanced social media addiction and created several physiological problems, sleep disorders, profound imbalance in psychological and interpersonal behaviors among all ages of people, particularly for the students (1,2-4). Therefore, the current study specifically hypothesized (hypothesis, H1 to H6) that several sociodemographic factors, eating and regular smoking habits, mental and interpersonal behaviors might strongly correlate with significant variation of BMI of the current university students in Bangladesh.

Hypothesis, H1: There might have a significant association of sociodemographic factors with BMI.

Hypothesis, H2: There might have a significant association of smoking with BMI.

Hypothesis, H3: There might have a significant association of eating large meals/snacks with BMI.

Hypothesis, H4: There might have a significant association of physical activity with BMI.

Hypothesis, H5: There might have a significant association of psychological behaviors (depression, anxiety, loneliness, sleep disturbance, suicidal thoughts) with BMI.

Hypothesis, H6: There might have a significant association of interpersonal behaviors (conflicts/arguments, physically or verbally abused) with BMI.

Moreover, the closure of educational institutions greatly impacts higher BMI that might be enhanced by several other factors, including unfavorable changes in eating habits like unhealthy consumption of junk foods, sleep disturbances, and less physical exercising and physical activity (5,6). Besides, eating behavior is significantly influenced by several social and cultural perspectives, and COVID-19 social lockdown has affected the current social life. Furthermore, it is a common belief that dietary choices and overall eating behaviors might strongly associate with several socioeconomic factors and altered social manners like eating alone vs. eating with others (7,8). Therefore, the following hypotheses (hypothesis, H7 to H12) regarding eating habits and physical activity need to be addressed at his current stage among university students in Bangladesh. Besides, to better understand the current study objective and hypothesis, a tentative conceptual framework might be sketched as **Figure S1**.

Hypothesis, H7: There might have a significant association of sociodemographic factors with eating habits.

Hypothesis, H8: There might have a significant association of smoking habit with eating habits.

Hypothesis, H9: There might have a significant association of sociodemographic factors with physical activity.

Hypothesis, H10: There might have a significant association of smoking habit with physical activity.

Hypothesis, H11: There might have a significant association of interpersonal behaviors (conflicts/arguments, physically or verbally abused) with physical activity.

Hypothesis, H12: There might have a significant association of eating habit with physical activity.

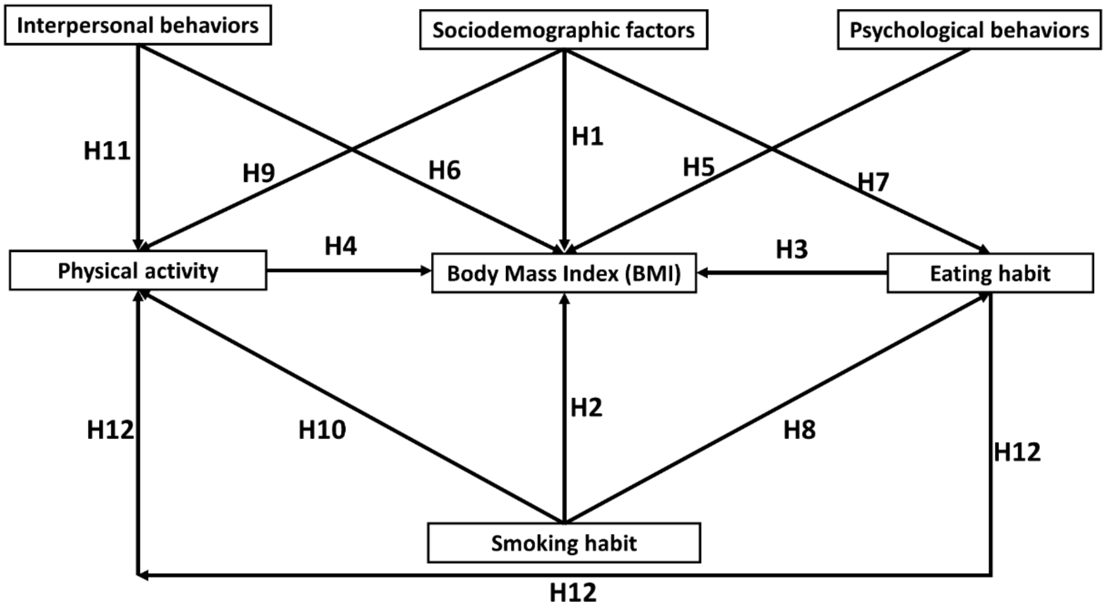

Figure S1. A tentative conceptual framework of the study. [H = Hypothesis]

Table S1. Frequency and percentage distribution of covariates potentially related with BMI, physical, psychological, and interpersonal behaviors following prolonged COVID-19 lockdown among university students in Bangladesh (N = 1,602).

Variables	Categories	Number (N)	Percentage (%)
Current BMI (kg/m^2) after prolonged COVID lockdown	Underweight	164	10.2
	Normal	533	33.3
	Overweight	684	42.7
	Obese	221	13.8
BMI (kg/m^2) before COVID lockdown	Underweight	190	11.9
	Normal	639	39.9
	Overweight	586	36.6
	Obese	187	11.7
Gender	Male	880	54.9
	Female	722	45.1
Age (years)	18 to < 22	465	29.0
	22-25	890	55.6
	> 25	247	15.4
Education level (level of schooling)	Lower grade	884	55.2
	Higher-grade	718	44.8
Current living area	Urban	969	60.5
	Rural	633	39.5
Monthly family income (BDT)	< 25,000	620	38.7
	25,000-50,000	642	40.1
	> 50,000	340	21.2
University type	Public	623	38.9
	Private	829	51.7
	Others	150	9.4
Smoking habit	Yes	227	14.2
	No	1375	85.8
Eating large meals or snacks	Increased	563	35.1
	Decreased	384	24.0
	Unchanged	655	40.9
Physical exercise	Increased	308	19.2
	Decreased	723	45.1

	Unchanged	571	35.6
Physical activity	Increased	274	17.1
	Decreased	903	56.4
	Unchanged	425	26.5
Sleep disturbance	Increased	701	43.8
	Decreased	386	24.1
	Unchanged	515	32.1
Feeling loneliness	Yes	1098	68.5
	No	504	31.5
Feeling depressed	Yes	1149	71.7
	No	453	28.3
Feeling anxious	Yes	1161	72.5
	No	441	27.5
Suicidal thoughts	Yes	340	21.2
	No	1262	78.8
Conflict/arguments with others	Yes	855	53.4
	No	747	46.6
Verbally or physically abused	Yes	459	28.7
	No	1143	71.3

Note: Lower-grade education = 1st/2nd/3rd year and higher-grade education = 4th/5th/Master's or above level students. Others category university type indicates various govt. college or private colleges under national university or medical college. Unchanged means the criteria is as like as before COVID-19 lockdown. BDT = Bangladeshi Taka; 1 USD = 84.48 BDT as of August 22, 2021.

References:

1. Al Hourani H, Alkhatib B, Abdullah M. Impact of COVID-19 Lockdown on Body Weight, Eating Habits, and Physical Activity of Jordanian Children and Adolescents. *Disaster Med Public Health Prep*. (2021) 16:1-9. doi: 10.1017/dmp.2021.48.
2. Robinson E, Boyland E, Chisholm A, Harrold J, Maloney NG, Marty L, Mead BR, Noonan R, Hardman CA. Obesity, eating behavior and physical activity during COVID-19 lockdown: A study of UK adults. *Appetite*. (2021) 156: 104853. doi: 10.1016/j.appet.2020.104853.
3. Islam MR, Jannath S, Moona AA, Akter S, Hossain MJ, Islam SMA. Association between the use of social networking sites and mental health of young generation in Bangladesh: A cross-sectional study. *J Community Psychol*. (2021) 49(7): 2276-2297. doi:10.1002/jcop.22675
4. Hossain MJ, Hridoy A, Rahman SMA, Ahmmed F. Major depressive and generalized anxiety disorders among university students during the second wave of covid-19 outbreak in Bangladesh. *Asia Pac J Public Health*. (2021) 10105395211014345. doi:10.1177/10105395211014345
5. von Hippel PT, Powell B, Downey DB, Rowland NJ. The effect of school on overweight in childhood: gain in body mass index during the school year and during summer vacation. *Am J Public Health.* (2007) 97(4): 696-702. doi:10.2105/AJPH.2005.080754
6. Pietrobelli A, Pecoraro L, Ferruzzi A, et al. Effects of COVID-19 Lockdown on Lifestyle Behaviors in Children with Obesity Living in Verona, Italy: A Longitudinal Study. *Obesity (Silver Spring).* (2020) 28(8):1382-1385. doi:10.1002/oby.22861
7. Herman CP, Roth DA, Polivy J. Effects of the presence of others on food intake: a normative interpretation. *Psychol Bull*. (2003) 129(6): 873-886. doi:10.1037/0033-2909.129.6.873
8. Cruwys T, Bevelander KE, Hermans RC. Social modeling of eating: a review of when and why social influence affects food intake and choice. *Appetite*. (2015) 86: 3-18. doi: 10.1016/j.appet.2014.08.035
